# A Scoping Review of Modifiable Risk Factors in Pediatric Onset Multiple Sclerosis: Building for the Future

**DOI:** 10.3390/children5110146

**Published:** 2018-10-26

**Authors:** Julie Pétrin, Maximillian D.J. Fiander, Prenitha Mercy Ignatius Arokia Doss, E. Ann Yeh

**Affiliations:** 1 Department of Rehabilitation Sciences, School of Rehabilitation Therapy, Queen’s University, Louise D Acton Building, 31 George St, Kingston, ON K7L 3N6, Canada; julie.petrin@queensu.ca; 2 Faculty of Medicine, Dalhousie University, Sir Charles Tupper Building, 5850 College Street, Halifax, NS B3H 4R2, Canada; max.fiander@dal.ca; 3 Department of Neurosciences, Faculty of Medicine, Université Laval, Pavillon Ferdinand Vandry, 1050, Medecine Avenue, Quebec City, QC G1V 0A6, Canada; prenitha-mercy.ignatius-arokia-doss.1@ulaval.ca; 4 Hospital for Sick Children, Division of Neurology, SickKids Research Institute, Neurosciences and Mental Health, University of Toronto, 27 King’s College Cir, Toronto, ON M5S 3H7, Canada

**Keywords:** multiple sclerosis, pediatric, lifestyle, risk factors, wellbeing, modifiable, scoping review

## Abstract

Knowledge of the effect of modifiable lifestyle factors in the pediatric multiple sclerosis (MS) population is limited. We therefore conducted a scoping review, following the framework provided by Arksey and O’Malley. Four databases were searched for pediatric MS and modifiable lifestyle factors using index terms and keywords, from inception to May 2018. All quantitative and qualitative primary articles were included and limited to English and full text. Of the 7202 articles identified and screened, 25 full-text articles were relevant to our objective and were included. These articles focused on diet obesity, physical activity, and sleep. In cross-sectional analyses, these lifestyle factors were associated with increased risk of pediatric onset MS (POMS), and increased disease activity. Diet, particularly vitamin D and vegetable intake, was associated with reduced relapse rate. Obesity was linked to increased risk of POMS, and physical activity was associated with reduced relapse rate and sleep/rest fatigue. Thus, available studies of lifestyle related outcomes in pediatric MS suggest specific lifestyle related factors, including obesity, higher vitamin D levels, and higher physical activity may associate with lower disease burden in POMS. Studies reviewed are limited by their observational designs. Future studies with longitudinal and experimental designs may further clarify the role of modifiable lifestyle factors in this population.

## 1. Introduction

Onset of multiple sclerosis (MS) prior to age 18 [1] occurs in 3–5% of cases of multiple sclerosis and has been increasingly recognized over the past 15 years [2,3]. Pediatric onset MS (POMS) patients experience a higher relapse rate [4], higher MRI disease burden [5], and disability at an earlier age than those with adult-onset MS [6]. They may experience significant decreases in quality of life and functional outcomes over their lifespan [7]. The etiology of the disease is multifactorial, with environmental and genetic factors likely interacting in the pathogenesis of the disease [8]. In particular, because of the young age of POMS patients, environmental factors may play an even more important role in POMS relative to adult-onset MS [9].

Both medical and patient communities have recognized the potential role lifestyle factors may play in modifying the disease course and severity of symptoms [10,11]. Furthermore, POMS patients and their parents are continually faced with making lifestyle decisions that may affect disease outcomes and quality of life [12]. Parents report encouraging their children to engage in “healthy” lifestyle choices, including making alterations to diet, physical activity (PA), and sleep behavior, which may (1) help parents feel more in control over the impact of the condition, and (2) allow them gain a more positive outlook on the future [13]. Unfortunately, even in the adult MS literature, there is insufficient evidence to make clinical recommendations regarding lifestyle modifications [10]. Improving the body of literature on modifiable lifestyle factors in pediatric MS with the goal of creating guidelines that will help POMS patients and their parents deal with these difficult decisions is needed [14,15].

Our objective in this manuscript is to summarize and identify gaps in current research on modifiable lifestyle factors and pediatric MS. Two questions guided this review: (1) Which modifiable lifestyle factors have been investigated in the context of POMS? And (2) which factors have been shown to play a role in the risk of POMS, disease course, or quality of life? 

## 2. Methods

We used the Arksey and O’Malley framework to guide this review. This framework includes the following steps: (1) Identifying the research question; (2) identifying relevant studies; (3) selecting studies; (4) synthesizing and interpreting data; and (5) collating, summarizing, and reporting results [16]. 

### 2.1. Search Strategy

The search was conducted within four databases (MEDLINE via Ovid, CINAHL Plus with Full Text, EMBASE and Cochrane library) and Google scholar to locate articles from inception to 7 May 2017. The search was updated on 25 May 2018. A search strategy was developed with the guidance of an information librarian in the MEDLINE database, which was adapted as needed for the subsequent databases. In order to ensure a good breadth of articles, an extensive list of subject headings and keyword searches were performed, varying slightly based on the term maps of each database, and truncations were used when appropriate. Subject headings and key words were used for multiple sclerosis, as well as pediatric or child or youth, and subsequently combined with the OR Boolean operator. Multiple additional searches were then conducted that were meant to capture a variety of lifestyle modifications and risk factors. These terms included, but were not limited to: Risk factors, lifestyle, modification, physical activity, health behavior, obesity, diet, nutrition, and sleep. These searches were then combined with the pediatric MS search, described above, with the AND Boolean. To ensure the search was comprehensive, hand searches of key citations reference list were performed. A total of 7202 citations were identified through database searches, and two additional citations were found through hand searches. 

### 2.2. Study Selection

**Figure 1:**
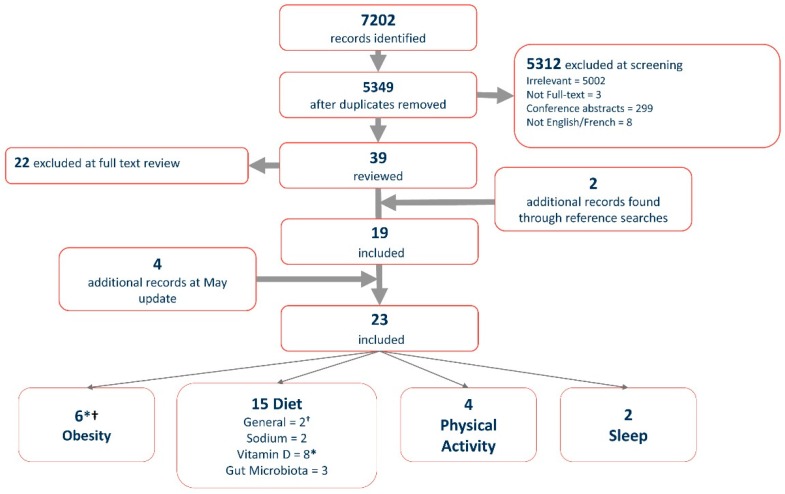
Modified PRISMA Chart. * Articles included in vitamin D and obesity sections: Brenton (2014), Gianfrancesco (2017), and Yamamoto (2018); ^†^ articles included in dietary factors and obesity sections: Pakpoor (2007): General diet and obesity.

All citations and abstracts were downloaded from each database and imported into EndNote, where duplicates were removed (*n* = 1853). Three of the Authors (JP, MF, and PAD) reviewed the titles and abstracts of the remaining 5349 citations to determine, which to include for full-text review. This screening process was guided by the following inclusion criteria: Articles pertaining to pediatric MS and lifestyle modifications, full-text, English, as well as these exclusion criteria: Conference abstracts, dissertations, protocols, editorials, letters to the editors, and reviews. All study designs were included in the analysis. All citations were screened by at least two authors. When disagreements occurred a third author was consulted. Please see Figure 1, an adapted PRISMA (Preferred Reporting Items for Systematic Reviews and Meta-Analyses) chart [17], which demonstrates the screening steps and citation totals. 

### 2.3. Data Charting and Analysis

Articles included in the analysis were reviewed by two authors and charted into an Excel data extraction file. A third author then verified the charted information to ensure accuracy. General study characteristics, including citation, modifiable lifestyle factor, study purpose and design, POMS population, sample size, POMS demographics, disease duration and level of disability, relevant outcomes, and main conclusions were recorded. The articles were subsequently grouped into four main modifiable lifestyle factor categories: Diet, obesity, physical activity, and sleep. Diet was furthered subdivided into general dietary factors, vitamin D intake, salt intake, and gut microbiota pertaining to dietary intake. Only two articles clearly focused on sleep as an outcome. Four of the articles addressed two modifiable lifestyle factors and thus were included in the two relevant categories [14,18,19,20] (see Figure 1). Each category was then further analyzed to determine the main conclusions drawn and the congruency of the findings. 

## 3. Results

The initial search resulted in 19 full-text articles that were relevant to our objective (see Figure 1). The search was updated on 25 May 2018, which led to the addition of 4 articles, therefore 23 articles were included in the present analysis [19,21,22,23]. Additional details related to demographics, disease-related information, and relevant results of all articles are available in the detailed evidence table (Supplementary Materials Table S1). 

### 3.1. Dietary Factors

Relationships between dietary factors and both POMS incidence and disease activity have been described in two studies [14,21]. The first [14], a cross-sectional study, evaluated whether higher consumption of any dietary factor was associated with pediatric MS (*n* = 312) when compared to healthy controls (*n* = 456). Enrolled patients, recruited from MS pediatric centers, were evaluated within 4 years of disease onset, with the 7-day recall questionnaire Block Kids Food Screener (BKFS) [24]. Dietary factors assessed included average caloric intake andconsumption of fats, proteins, carbohydrates, sugars, fruit, vegetables, dairy, fiber, iron, and beverages [24]. The only dietary factor that reached significance after covariate adjustment was iron. Iron intake below recommended amounts was associated with risk of POMS (*p* < 0.01), suggesting that maintaining appropriate iron levels may reduce the risk of POMS. The same group also [21] evaluated dietary intake at an early time point post-diagnosis and its relationship to relapse rate in POMS. Participants (*n* = 219) had a mean disease duration prior to study enrollment of 0.9 years (SD = 0.9) and median follow-up of 1.8 years (range = 0.1–4.1). The clinical endpoint was time to first relapse following study enrollment. After covariate adjustments only associations between relapse risk and saturated fat and vegetable intake remained significant. Specifically, a 10% increase in caloric intake from saturated fats was associated with a tripling of relapse risk (*p* = 0.014). Conversely, a 10% increase in caloric intake from vegetables cut relapse risk in half (*p* = 0.043). 

Together these studies suggest associations between dietary factors, such as saturated fats and vegetable intake and relapse rate in POMS, and increased risk of POMS in those with dietary iron levels below recommended levels. Limitations of the studies include recall bias, as data was derived from a 7-day recall questionnaire, and referral bias, as patients were selected from specialized MS centers. As well, potential confounders, such as exercise and obesity were not controlled for. Finally, the BKFS was administered solely at enrollment therefore did not account for the possibility of dietary changes following diagnosis. 

#### 3.1.1. Dietary Micronutrients

##### Sodium

Two studies from the same group [25,26] investigated the effects of dietary sodium intake in individuals with POMS. The first used a case-control design to investigate whether individuals consuming higher amounts of sodium were at a higher risk of developing POMS [26]. The second, a cohort study (*n* = 174), aimed to determine whether high consumption of dietary sodium was associated with time to relapse in POMS patients [25]. Time to relapse was calculated from time of enrollment to the end of the study (4 years). Again, the information source was the BKFS in both studies. The first study, with 170 POMS cases and 331 controls, found no association between POMS and sodium intake [26]. Similarly, the second study, found no association between dietary intake of sodium exceeding the daily recommended allowance and time to next relapse [25]. Neither study found any evidence for a relationship between higher dietary sodium intake on onset or relapse risk in pediatric MS. As with the previously described publications, potential limitations include recall and referral bias, as well as lack of controlling for confounders, such as vitamin D intake and exercise. Furthermore, the relatively small number of participants (174 patients) may have limited the power of the study to show differences between patients and controls. 

##### Vitamin D

Consistent results in 7 studies suggest that lower vitamin D levels may confer higher risk of MS diagnosis and disease activity in youth. Vitamin D deficiency or insufficiency is highly prevalent in POMS patients. Brenton et al. (2014), investigated the prevalence of vitamin D deficiency and its associated factors on POMS (*n* = 24) versus adult-onset MS (*n* = 92) using a retrospective cohort design [20]. Both POMS and adult-onset MS had similarly high rates of vitamin D deficiency (50%) and insufficiency (84%), with no significant differences between the two patient populations (*p* = 0.81 and *p* = 0.91, respectively) [20]. Two additional descriptive cohort studies of POMS patients (*n* = 60 [19] and *n* = 111 [23]) added to this growing evidence by demonstrating that 63% [19] and 68.5% [23] of their POMS cohort had low 25(OH)-vitamin D (below 30 ng/mL). More conclusively, Banwell (2011) [27] examined whether serum vitamin D status at the time of first CNS demyelination was associated with likelihood of later diagnosis with POMS in a prospective cohort study. Serum 25-hydroxyvitamin D was measured within 40 days of symptom onset in consecutive patients (*n* = 302), from pediatric centers. Participants were reassessed every 3 months for the first year and yearly subsequently [27]. Baseline vitamin D status was associated with risk of developing POMS, specifically, a 10 nmol/L decrease in vitamin D was associated with an increased risk of POMS (*p* = 0.006). 

In addition to conferring risk of POMS, lower vitamin D levels have also been associated with higher relapse rate in POMS. Mowry et al. (2010) [28], evaluated the relationship between serum vitamin D at the time of diagnosis and relapse rate in POMS. Patients with POMS (*n* = 110) recruited from Pediatric MS clinics were followed and the number of relapses over the follow-up period (median 1.7 years, IQR 0.2–4.0) was measured to determine the relapse rate from enrollment to study end. After covariate adjustment, each 10 ng/mL decrease in baseline serum 25-hydroxyvitamin D status was associated with a 33% increase in risk of relapse (*p* = 0.02) [28]. Another study by this group [29] demonstrated that the role of vitamin D in POMS relapse rates is, at least in part, genetically mediated by examining genetic predictors of relapse risk in POMS patients (*n* = 181). Consistent with Mowry (2010), their results showed a 10 ng/mL increase in serum 25-Hydroxyvitamin D was associated with a decreased risk of relapse, however this only reached significance if the patient had at least one copy of HLA-DRB1*15:01 or 15:03 allele *(p* = 0.001) [29].

Further evidence of the importance of vitamin D in POMS comes from a larger study led by Gianfrancesco [18] that sought to investigate the relationship between vitamin D status and risk of POMS. This study employed Mendelian randomization, a study design that uses single nucleotide polymorphisms (SNPs) known to be associated with a particular risk factor (vitamin D in this case) to establish a more causal link between the risk factor and the outcome. Genetic vitamin D variants applied to two large datasets from the US (394 cases, 10,875 controls) and Sweden (175 cases, 5376 controls) showed that higher levels of serum vitamin D, represented by a computed genetic risk score for 3 genetic variants associated with higher levels of serum vitamin D, were also associated with reduced risk of POMS (*p* = 0.002). This study had a large sample size, however, generalizability of the study may be limited, because only a single racial group (non-Hispanic white) was included.

Finally, associations between vitamin D and MS in youth may be mediated by infection and genetic status. Mowry et al. (2011) examined the relationship between vitamin D status and antibodies to common childhood viruses in POMS (*n* = 120) and Clinically Isolated Syndrome (CIS) patients (*n* = 20). No clear associations between vitamin D status and viral antibody titers were found, however some differences were present. For instance, individuals with MS or CIS, higher levels of vitamin D were associated with higher antibody levels of CMV and HSV-2, which was not observed in healthy controls [30].

#### 3.1.2. Gut Microbiome

Three small studies from the same group examined the role of the gut microbiota in POMS youth, suggesting (1) a possible association between pediatric MS and changes in the gut microbiome; and (2) that changes within the gut microbiome and immune relationships may increase the risk of relapse [9,31,32].

The first [32], a case-control study, examined the gut microbiota in youth with POMS (*n* = 18) compared to age and sex-matched healthy controls (*n* = 17). Significant differences were found at the level of the phylum. Individuals with POMS had 2.5 times higher presence of Actinobacteria compared to controls. There was also a marked increase in pro-inflammatory Desulfovibrio genera, from the heritable Christensenellaceae family, and a marked depletion in anti-inflammatory members of Clostridiales order (*p* < 0.0000005, false discovery rate adjusted *p* values of <0.05 were considered significant). Microbial genes involved in glutathione metabolic pathway were more abundant in cases versus controls (Mann-Whitney, *p* = 0.017). Together these results suggest that early stages of pediatric MS involve a dysregulation of gut microbiota that promotes a more pro-inflammatory gut microbiota. 

The second study [9] examined the association between the gut microbiota and subsequent relapse rate in 17 POMS youth, followed for a mean of 19.8 months. The results demonstrated that low levels or an absence of Fusobacteria (*p* = 0.001), higher levels of Firmicutes (*p* = 0.003), and a presence of Archaea Euyarchaeota (*p* = 0.037) were associated with a shorter time to relapse. The absence of Fusobacteria phylum was associated with a 76% risk of an earlier relapse (*p* = 0.024), which remained significant after covariate adjustment.

The final study [31] explored the association between gut microbiota and host immunological markers of POMS cases (*n* = 15) compared to age and sex-matched healthy controls (*n* = 9). The results suggested an association between host blood immune marker profiles in children with and without MS and gut microbiota composition. There were measurable differences between POMS and control children in microbiota-immune relationships early in the disease course. For instance, richness, the number of unique taxa identified, was positively correlated with Th17 for cases (r = 0.665, *p* = 0.018), and not with controls (r = −0.644, *p* = 0.041). Bateroidetes inversely correlated with Th17 for POMS cases (r = −0.719, *p* = 0.008, not controls (r = 0.320, *p* = 0.401). Lastly, a strong positive association was found between Fusobacteria abundance and Tregs in controls (r = 0.829, *p* = 0.006), not cases, r = −0.069, *p* = 0.808). The two studies above suggest that alterations in the gut microbiota may have effects on disease activity MS and T-cell expression. Further studies are needed to confirm these findings.

### 3.2. Obesity

There is growing interest and evidence supporting associations between POMS and childhood obesity. We identified 6 studies [14,18,20,33,34] describing associations between obesity on POMS risk. Three studies reported high rates of obesity in POMS patients. The first, a descriptive retrospective single center cross-sectional study of youth with POMS [19], reported that 49% of their cohort were overweight or obese, as determined by Body Mass Index (BMI) [19]. Pakpoor et al. [14] (described in detail above) demonstrated that POMS youth had higher BMIs than controls (*p* < 0.001), while Brenton et al. (2014) (described in more detail above) sought to investigate the prevalence of obesity, as measured by BMI, in POMS versus adult onset MS. This study reported that POMS individuals were significantly more obese than their adult-onset counterparts (*p* = 0.02) [20]. 

Gianfrancesco et al. [18] (described in more detail above) furthered these results by demonstrating a causal association between increased BMI associated SNPs and the risk of POMS (*p* = 0.01), using mendelian randomization [35]. This association was also found by two further studies and was extended by demonstrating that this increased risk may be dependent on pubertal changes [34,35]. 

Langer-Gould et al. (2013) aimed to determine if childhood obesity, as measured by BMI, was a risk factor associated with developing POMS or CIS [33]. In this case-control study, cases of new-onset POMS or CIS (*n* = 75) and controls (*n* = 913,097) were identified in the Kaiser Permanente Southern California system. Increased BMI was associated with increased risk of POMS/CIS in girls (*p* < 0.005), but not boys. This increased risk was found in children with onset between the ages of 12 and 18, but not in children with earlier onset, suggesting an interaction with puberty and sex hormones. Chitnis et al. (2016) sought to further explore the relationships between BMI and pubertal measures on the risk and age of onset of POMS [34]. Cases (*n* = 254) were compared to controls (*n* = 420), which were recruited from pediatric clinics. Percentile BMI was calculated within one year of disease onset. Tanner staging was also performed using sexual maturity measurements. Similar to Langer-Gould (2013), an association between BMI and POMS was found. Increased BMI was associated with increased risk of POMS in post-pubertal girls (*p* = 0.009), but not in pre-pubertal girls. Sample size was insufficient to assess pre- and post-pubertal boys separately, but assessed together, high BMI also increased risk of POMS (*p* = 0.011). Furthermore, age of onset was 0.91 years earlier in overweight or obese girls (*p* = 0.022). A strength of this study was that it directly investigated pubertal measures. However, a larger sample size may have allowed elucidation of the role of puberty in boys with MS and aided in dissociating the inter-related factors of BMI, puberty onset, and risk of POMS. 

### 3.3. Physical Activity

The relationship between physical activity and POMS outcomes has been evaluated in four studies [22,36,37,38]. In these studies, POMS patients were compared to monophasic demyelinating syndrome (mono-ADS) patients [36,37], healthy control participants [37,38], and reference values from healthy children [22]. 

In a case-control study of consecutively enrolled patients from a pediatric MS center, Grover (2015) [36] evaluated the relationship between PA and disease burden in individuals with POMS (*n* = 31) versus mono-ADS (*n* = 79). PA was assessed using the Godin Leisure-Time Exercise Questionnaire (GLTEQ), a validated questionnaire for assessing PA in pediatric populations [39]. The study found that POMS patients engaged in less strenuous activity than mono-ADS patients (*p* = 0.0012). Additionally, only 45.2% of POMS patients participated in strenuous activity as compared to those with mono-ADS (82.3%, *p* = 0.0003). The authors also reported that PA levels were negatively correlated with depression, as measured by the Center for Epidemiological Studies Depression Scale for children (CES-DC) [40] and fatigue, as measured by the Varni PedsQL MFS [41], meaning that patients with higher levels of PA had lower levels of both fatigue and depression. Lastly, PA levels were associated with clinical disease activity. Higher strenuous PA was correlated with lower T2 lesion load and annualized relapse rate (r = −0.66, *p* = 0.006). 

In a second paper by the same group, Grover (2016) [37] examined PA levels in patients with POMS, mono-ADS, and healthy controls. Barriers and facilitators to physical activity were also examined. PA goal setting and self-efficacy as measured by the Exercise Goal-Setting Scale (EGS) [42] and the Physical Activity Self-Efficacy Scale (PASES) [43], respectively, were associated with more engagement in vigorous PA, as assessed by accelerometry and GLTEQ in POMS participants. Mirroring the results of Grover et al. (2015), POMS patients engaged in less moderate (*p* = 0.009) and strenuous PA than patients with mono-ADS and healthy controls (*p* = 0.048). Further, a lower proportion of POMS patients (65%) participated in strenuous activity than did the other two groups (85, 89%; *p* = 0.02). PASES and EGS were positively associated with PA levels. Both of these correlates of PA could be modified to increase PA participation.

A third study by this group [38], aimed to validate the GLTEQ relative to accelerometry in youth with MS. They reported strong correlations between GLTEQ and accelerometry data indicating that the GLTEQ may be useful as an alternative to accelerometry in future research. Lastly, Toussaint-Duyster (2017) [22] sought to evaluate the interactions between fatigue (PedsQL-MFS) and maximal exercise capacity, as measured by the Bruce Protocol [44], as well as interactions with other factors, including motor skills, and sports participation. They found decreased exercise capacity (*p* < 0.001) and motor skills in POMS patients (Mean SDS = 13 (35.1), *p* < 0.001), particularly in balance subscales (*p* < 0.001). Further, decreased exercise capacity correlated with decreased participation in organized sports (r = 0.365, *p* = 0.034). 

### 3.4. Sleep

Sleep disorders may be associated with poor outcomes in adolescents, including daytime fatigue, impaired social and emotional functioning, and neurocognitive issues [45]. Only 2 published studies have directly investigated sleep in individuals with POMS [46,47]. Zafar et al. (2015) performed a case-control study to determine whether individuals with pediatric MS (*n* = 30) experience more sleep disturbances, daytime sleepiness, and fatigue than age-, sex-, and race-matched controls (*n* = 52). Interestingly, individuals with POMS were found to have better sleep hygiene, particularly in relation to sleep stability (*p* = 0.0052), greater frequency of adherence to a usual sleep time throughout the week, and also less daytime sleepiness than controls (*p* = 0.0061) [46]. A second, qualitative study examined experiences of fatigue in youth with POMS (*n* = 15) and their parents (*n* = 13) through in-depth semi-structured interviews [47]. Interviews were audio-recorded and transcribed for thematic analysis. In interviews, children with POMS described napping as occurring in association with daytime fatigue, which in turn was postulated to disrupt their sleep patterns and lead to poor sleep quality. This study also highlighted differences between the importance parents placed on fatigue as compared to their children with POMS, with parents perceiving fatigue to be worse than the youth with POMS. 

## 4. Discussion

Information on lifestyle modification is relevant to individuals with POMS and their families. This scoping review highlights that this field is in its nascent stages, with the first publication on this topic occurring in 2009. This review emphasizes the small number of observational studies that have been conducted on modifiable lifestyle factors and their relationship to risk of POMS or POMS disease activity. 

In total, employing a broad search strategy, only 23 publications satisfied our inclusion criteria. While the number of studies retrieved was small, they covered a wide range of lifestyle issues. The greatest number of publications focused on dietary factors and their relation to risk of POMS onset or disease activity (*n* = 15). The most studied dietary factor was vitamin D (*n* = 8), which was consistently found to have a protective role. The other dietary studies examined a range of micro- and macronutrients, including salt, iron, and saturated fats. Three of these studies examined gut microbiota in the context of POMS. These studies consistently reported dysregulation of gut flora in POMS that was associated with risk of relapse and immunological markers. These findings suggest the importance of the emerging field of gut microbiota in POMS. Studies examining the role of physical activity and POMS risk and disease activity were also found (*n* = 4). Physical activity was associated with decreased disease burden and decreased fatigue in POMS. Closely related to these findings, associations were also found between obesity and risk of POMS. The abovementioned findings have relevance for future work in this area: Differences in dietary factors, including vitamin D may affect the composition of the gut microbiota [48,49], a possible etiology of gut dysbiosis that has been associated with disease progression in experimental models of MS [49,50]. Finally, studies reviewed suggested that sleep hygiene may be key in managing daytime sleepiness in youth with POMS. Together these results suggest that altering diet and exercise patterns could be beneficial in reducing disease activity both directly, and indirectly, via a decrease in BMI and potentially by altering gut microbiota. 

Research in this field remains a challenge, as pediatric MS is a relatively rare disease, leading to difficulties in gaining large enough sample sizes to appropriately power studies: Collaborative multi-site studies are therefore a necessity in the future. Future research efforts should be oriented towards longitudinal assessments, which will allow for greater clarity regarding directionality of these associations. Furthermore, future studies should ensure that psychosocial outcomes, including quality of life are included along with disease-related outcomes. However, unlike pharmaceutical interventions, interventions focused on lifestyle must take factors that may affect health behaviors into account in trial design. As previous work has shown, interventions based on the tenets of social cognitive theory, with a focus specifically on goal setting and self-efficacy, may be effective in promoting behavior change in this cohort [37]. Finally, future research is needed to examine the underlying mechanisms whereby lifestyle factors lead to changes in disease activity or outcomes. 

## Supplementary Materials

The following are available online at http://www.mdpi.com/2227-9067/5/11/146/s1, Table S1: Data Extraction.
