# Supplementary material for: A Scoping Review of Modifiable Risk Factors in Pediatric Onset Multiple Sclerosis: Building for the Future
Source: Children (Basel). 2018 Oct 26;5(11):146. doi: 10.3390/children5110146 (PMC6262383; doi:10.3390/children5110146)
Supplement: Supplementary file 1 [file children-05-00146-s001.pdf]

**Author, Year**	**Purpose, Aim or Questions**	**Study Design**	**Total Sample (N)**	**n per group**	**Age** *(mean and SD)*	**% Female**	**Disease Duration** *(mean SD, yrs)*	**Relevant Outcomes**	**Relevant Findings**
Pakpoor et al. 2017 [14]	To determine the association between dietary factors and POMS patients.	Case-control	768	**Case** 312 **Control** 456	**Case** 15.1 (3.3) **Control** 14.4 (3.7)	**Case** 62.5 **Control** 52.9	10.8 (10.2)	1. **Block Kids Food Screener (BKFS)** 2. **BMI**	• Covariate adjusted model (age, sex, ethnicity, race, BMI, and socioeconomic status) showed that iron levels below RDA was associated with an increased risk of POMS (odds ratio (OR) = 1.80, 95% confidence intervals (Cl) 1.24-2.62, $p < 0.01$). • Individuals with POMS had higher BMIs (M=25.3, SD 7.1) than controls (M=22.1, SD 5.7; p < 0.001).
Azary et al. 2017 [21]	To investigate the effects of diet on relapse rate in POMS youth.	Prospective cohort	219	N/A	15.1 (3.3)	61.2	0.9 (0.9)	1. **BKFS** 2. **Time to relapse** (post enrollment to end of study)	• After adjusting for age, sex, race, ethnicity, disease duration, BMI, total energy intake, use of disease modifying therapies, and baseline vitamin D levels, only vegetable and saturated fat intake were associated with relapse risk in POMS. A 10 % increase in caloric intake of saturated fats led to a tripling of the relapse risk (adjusted hazard ratio (HR): 3.22, 95% CI 1.26 – 8.17, p=0.014) Whereas, the same increase in caloric

									intake in vegetables cut relapse risk in half (adjusted HR: 0.53, 95% CI 0.28 – 0.98, *p*=0.043).
McDonald et al. 2016 [26]	To investigate the association between dietary salt intake and POMS risk.	Case-control	501	**Case** 170 **Control** 331	**Case** 15.2 (3.5) **Control** 14.0 (3.7)	**Cases** 62.9 **Control** 48.6	1.0 (1.2)	1. **BKFS**	• No association was found between risk of POMS and higher salt intake (OR=1.00, 95% CI 0.98, 1.02; *p*=0.93) or excess salt intake (OR=1.05,95% CI 0.67,1.64; *p*=0.84).
Nourbakhsh et al. 2016 [25]	To determine if dietary salt intake is associated with time to relapse in individuals with POMS and CIS.	Prospective cohort	174	N/A	15.0 (3.3)	64.9	Not reported	1. **BKFS** 2. **Time to relapse** (post enrollment to end of study)	• No associations were found between sodium intake and time to next relapse. Patients with higher sodium intake had a HR of 0.69 (95% CI 0.37 to 1.30, *p*=0.25) whereas patients with low intake had an HR of 1.37 (95% CI 0.74 to 2.51, *p*=0.32).
Brenton et al. 2014 [20]	To determine the prevalence of vitamin D insufficiency and deficiency in POMS and adult-onset MS.	Retrospective cohort	116	**POMS** 24 **Young Adult-onset MS** 33 **Adult-onset MS** 59	***Age of onset*** **POMS** 14.6 range (7-17) **Young Adult-onset MS** 19.7 range (18-21) **Adult-onset MS** 28.9	**POMS** 71 **Young Adult-onset MS** 73 **Adult-onset MS** 69	Not reported	1. **Levels of 25-hydroxyvitamin D3**: (within 12 months before or after the established diagnosis was made) *Insufficient less than 30 ng/ml; deficient less than 20 ng/ml* 2. **BMI (**within a 3 month period of the vitamin D	• No differences were found between age groups, however both groups had high percentage of individuals that were vitamin D deficient (50%) and insufficient (84%).

					range (22-57)			level draw)<br>*Overweight*: *BMI above 25-30*<br>*Obese: BMI above 30*	
Yamamoto et al. 2018 [19]	To describe the POMS patient population from one center over 13 years.	Retrospective cohort	60	N/A	15.7 (2.9)	68	Not reported	1. **Annualize relapse rate**<br>2. **25(OH)-vitamin D**<br>*Low levels were set at below 30 ng/ml*<br>3. **BMI**	• 63% of individuals with POMS in this study had low serum vitamin D levels.<br>• 49% of the cohort were overweight or obese, as determined by the BMI.
Yilmaz et al. 2017 [23]	To describe the features of POMS youth in Turkey.	Retrospective cohort	193	193	13.47 (2.88)	63.7	Not Reported	1. **Serum 25-hydroxyvitamin D levels**	• 68% of the Turkish cohort had low serum vitamin D levels.
Banwell et al. 2011 [27]	To determine the impact of vitamin D insufficiency, at first demyelinating event, on risk of POMS development.	Prospective cohort	302	**Case**<br>63<br>**Mono-ADS)**<br>239	***Age at onset***<br>**Case**<br>12.0 (3.8)<br>**Mono-ADS**<br>8.85 (4.5)	**Case**<br>*65*<br>**Mono-ADS**<br>*48*	Not reported	1. **Serum 25-hydroxyvitamin D** (samples taken within 40 days of symptom onset and categorized seasonally)<br>2. **MS diagnosis**	• Over half (68%) of the participants had serum vitamin D levels lower than 75 nmol/L.<br>• A 10 nmol/L decrease in vitamin D was associated with an increased risk of POMS (HR=0·89, 95% CI 0·80–0·98, p=0.006).

Mowry et al. 2010<br>[28]	To investigate if vitamin D status, is associated with relapse rate in POMS.	Prospective cohort	110	N/A	15.0 (3.0)	65%	Mean: 1 IQR (0.1-8.3)	1. **Relapse rate** (number of relapses from blood draw to last follow-up)<br>2. **Serum 25-hydroxyvitamin** D (samples were deseasonalized and stratified by race and ethnicity)	After adjusteing for race, season and ethnicity, baseline vitamin D status was associated with a 33% increase in risk of relapse with each 10 ng/ml decrease (incidence rate ratio 0.66, 95% CI 0.46 – 0.95, *p*= 0.02).
Graves et al. 2016<br>[30]	To determine if genetic ancestry, sex, HLA-DBRB1*14, vitamin D levels, and non-HLA GRS are associated with rate of relapse in POMS youth.	Prospective cohort	181	N/A	***Age at onset***<br>13.1 (4.2)	65.8	Not reported	1. **Levels of 25-hydroxyvitamin D3** (baseline serum samples)<br>2. **Annualized relapse rate**<br>3. **HLA-DRB1* 15.01 or 15.03 (**Presence)	• A 10ng/ml higher level of vitamin D only led to decreased relapse risk if individuals had at least one copy of either identified allele (HR = 0.73, 95% CI = 0.60–0.89, *p*= 0.001), while adjusting for DMT and sex.
Gianfrancesco et al. 2017<br>[18]	To estimate the causal association between low serum vitamin D levels, high BMI, and POMS using genetic risk scores.	Case-control with mendelian randomizatio n	16,820	**US Case** 394<br>**US Control** 10875<br>**Sweden Case** 175<br>**Sweden control** 5376	***Age at Onset***<br>**USA** 14.05 (3.3)<br>**Sweden** 14.91 (2.67)	**USA** 75.0<br>**Sweden** 71.4	Not reported	1. **Vitamin D** Genetic Risk Factor (GRS)<br>2. **BMI** GRS	• SNPs that were associated with higher levels of serum vitamin D were associated with a reduced risk of POMS (OR 0.72 95% CI 0.55-0.9, *p*= 0.02).<br>• SNPs associated with obesity were associated with increased risk of POMS (OR: 1.17, 95% CI 1.05, 1.30; p = 0.01).

									• BMI and vitamin D levels act independently to alter disease risk in POMS.
Mowry et al. 2011 [29]	To investigate if vitamin D status is associated with antibody levels to common childhood viruses and whether these associations differ based on POMS status.	Retrospective cohort	140	**POMS** 120 **CIS** 20	**POMS** 15.0 (3.5) **CIS** 13.8 (3.9)	**POMS** 63 **CIS** 60	Mean:1.2 IQR (0.1-8.3)	1. **Levels of 25-hydroxyvitamin** D3 in baseline serum samples 2. **Viral assays** Batched EBV viral capsid antigen (VCA), cytomegalovirus (CMV), and herpes simplex virus (HSV)-1 and -2 assays (IgG)	• POMS/CIS individuals with vitamin D sufficiency (over 30 ng/mL), had higher antibody levels to Epstein-Barr nuclear antigen-1 (coefficient=0.49, 95% CI 0.02, 0.97, *p*=0.043) than controls. This sufficiency was also associated with higher CMV antibody levels in POMS/CIS subjects (coefficient 1.04, 95% CI 0.36, 1.73, *p*=0.004) but lower CMV antibody levels in controls (coefficient −1.10, 95% CI −2.44, 0.25, p=0.11). • Higher vitamin D levels were also associated with higher titers to HSV-2 in MS/ CIS patients (coefficient 0.05, 95% CI 0.01, 0.09, *p*=0.030) but not controls.
Tremlett et al. 2016 [33]	To explore the gut microbiota in early onset POMS compared to controls.	Case-control	35	**Case** 18 **Control** 17	**Case** 12.5 (4.44) **Control** 13.5 (3.08)	**Case** 56 **Control** 53	10.6 months (6.34)	**1. Alpha diversity** expressed as evenness, richness and faith phylogenic diversity metric **2. Beta diversity** measured using	• Significant differences were found at the levels of the phylum. • MS cases had a significant enrichment in relative abundance for members of the *Desulfovibrionaceae* and depletion in

								Canberra distance matrix	*Lachnospiraceae* and *Ruminococcaceae* (all P and q < 0.000005). Microbial genes involved in glutathione metabolic pathway were more abundant in cases versus controls (Mann-Whitney, *p*=0.017).
Tremlett et al. 2016 [31]	To explore the association between gut microbiota in early POMS and relapse risk.	Prospective cohort	17	17	12.5 (4.57)	59%	10.3 months (6.6)	1. **Relapse rate -** (determined via structured forms and chart review by abstractors)<br>2. **Gut Microbiome Profiles**	• Low levels or an absence of *Fusobacteria* (*p*=0.001, log-rank test), higher levels of *Firmicutes* (*p*=0.003), and a presence of *Archaea Euyarchaeota* (*p*=0.037) were associated with a shorter time to relapse.<br>• Absence of *Fusobacteria* was associated with a 76% (95% CI: 55%-90%) risk of an earlier relapse (HR=3.2, 95% CI; 1.2-9, p = 0.024), which remained significant after covariate adjustment (age and immunomodulatory drug exposure).
Tremlett et al. 2016 [32]	To explore associations between the gut microbiota and blood immunological markers POMS cases early in their disease course compared to	Case-control	24	**Case** 15 **Control** 9	**Case** 11.9 (4.64) **Control** 13.8 (3.19)	**MS** 53 **Control** 78	10.0 months range 2–23 months	1. **Microbiota Diversity**<br>2. **Phylum level abundances**<br>3. **Immune markers** *Treg frequency and intracellular cytokine*	• Measurable differences were found between blood immune host markers and gut microbiota between cases and controls.

	healthy controls.							*production by T-cell*	
Langer-Gould et al. 2013 [34]	To examine if childhood obesity is associated with the risk of developing POMS or CIS.	Case-control	913172	**Case**<br>75<br>**Control**<br>913,097	**Case**<br>*2-11*<br>28%<br>*12-18*<br>72%<br>**Control**<br>*2-11*<br>49.1%<br>*12-18*<br>50.0%	**Case**<br>54.7<br>**Control**<br>49.7	Not reported	1. **BMI**<br>*WHO definition for weight classes*	• 50.7% of cases were overweight or obese.<br>• Increased BMI was associated with increased risk of POMS/CIS in girls (OR = 3.76, 95% CI = 1.54 - 9.16, $p < 0.005$) but not boys.
Chitnis et al. 2016 [35]	To determine the relative contributions of BMI and pubertal measures for risk and age of onset of pediatric MS.	Case-control	674	**Case**<br>254<br>**Control**<br>420	**Case**<br>14 (3.4)<br>**Control**<br>14 (3.7)	**Case**<br>63<br>**Control**<br>49	Less than 4 years	**1. BMI**<br>(within 1 year of MS onset)<br>*Obesity defined as BMI above the 85th or 95th percentiles for age.*<br>2. **Sexual maturity measurements: Tanner Staging**	• Increased BMI was associated with increased risk of POMS in post-pubertal girls (adjusted OR = 1.60, 95% CI: 1.12–2.27, p=0.009) but not in pre-pubertal girls.<br>• Sample size was insufficient to assess pre- and post-pubertal boys separately, but assessed together, high BMI also increased risk of POMS (adjusted OR = 1.43, 95% CI 1.08–1.88, p=0.011).<br>• Age of onset was 0.91 years earlier in overweight or obese girls (95% CI: 0.14–1.67, p=0.022).

Grover et al. 2015 [38]	To examine the association between physical activity (PA) and MS disease activity, depression, and fatigue in children with MS and monophasic acquired demyelinating syndrome (mono-ADS).	Cross-sectional cohort	110	**Case** 31 **Mono-ADS** 79	**Case** 15.91 (2.36) **Mono-ADS** 13.91 (4.43)	**Case** 81 **Mono-ADS** 54	*Median (IQR)* **Case** 1.64 (4.22) **Mono-ADS** 3.06 (5.03)	1. **Annualized relapse rate** 2. **Fatigue** Varni PedsOL multidimensiona l fatigue scale (PedsQL MFS) 3. **Depression** Centre for Epidemiological studies of Depression Scale for Children (CES-DC) 4. **Physical activity** Godin Leisure-Time Exercise Questionnaire (GLTEQ) 5. **Disease burden** T1 and T2 lesion load on MRI	• POMS youth engage in less strenuous physical activity (median 0.0, IQR 27.0) than mono-ADS patients (median 27.0, IQR 36.0; *p*=0.0012). • Only 45.2% of POMS patients participated in strenuous activity as compared to those with Mono-ADS ADS (82.3%, *p*=0.0003). • PA levels were negatively correlated with depression and fatigue. • Higher strenuous PA was correlated with lower T2 lesion load (r=-0.66, *p*=0.006) and ARR (r=-.66, p=0.006).
Grover et al. 2016 [39]	To examine PA levels in youth with POMS and mono-ADS, compared with healthy controls and to determine factors that lead to engaging in PA.	Case control	106	**MS** 27 **Mono-ADS** 41 **Control** 37	*Median (IQR)* **MS** 16.0 (4.0) **Mono-ADS** 14.0 (4.0) **Control** 15.0 (3.0)	**MS** 67 **Mono-ADS** 46 **Control** 68	*Median (IQR)* **MS** 2.0 (2.0) **Mono-ADS** 4.0 (7.0)	**1. Physical Activity** GLTEQ 2. **Fatigue** PedsQL MFS 3. **Depression** CES-DC 4. **Self-efficacy** Physical Activity Self-Efficacy Scale (PASES) 5. **Goal Setting** The Exercise Goal-Setting	• PA goal setting was associated with engagement in vigorous PA, as assessed by accelerometry (*p*=0.0003), GLTEQ (*p*=0.006) in POMS patients. • PA self-efficacy was associated with engagement in vigorous PA, as assessed by accelerometry (p=0.02) in POMS patients. • POMS patients engaged in less moderate (*p*=0.009)

								Scale (EGS)<br>6. **Sports Participation** short questions about sport engagement	and strenuous (*p*= 0.048) PA than patients with mono-ADS and healthy controls.<br>• A lower proportion of POMS patients (65%) participated in strenuous activity than did the other two groups (85-89%; *p*= 0.02)<br>• PASES and EGS were positively associated with PA levels
Kinnett-Hopkins et al. 2016 [40]	To examine the validity of GLTEQ as a measure of PA in POMS.	Validity study	72	**Case**<br>27<br>**Control**<br>45	*Median (IQR)*<br>**Case**<br>15.73 (3.2)<br>**Control**<br>14.76 (3.8)	**Case**<br>66.7<br>**Control**<br>66.7	*Median (IQR)*<br>2.03 (2)	**1. Physical activity**<br>GLTEQ and accelerometer data	• A strong correlation was found between GLTEQ and accelerometer for measuring physical activity in individuals with POMS. This positive correlation reached significance for vigorous PA (r=0.736, *p*=0.001), and nearly met significance from moderate (r=0.319, p=0.053).
Toussaint-Duyster et al. 2017 [22]	To examine the interaction between exercise capacity, motor performance, neurological	Cross-sectional	38	**MS**<br>22<br>**Post-ADEM**<br>16	*Median (IQR)*<br>**MS**<br>14 (13-15)<br>**Post-ADEM**	**MS**<br>82<br>**Post-ADEM**<br>44	*Median (IQR) (months)*<br>**MS**<br>10.2 (4.6-21.5)<br>**Post-ADEM**	1. **Fatigue**<br>PedsQL MFS<br>2. **Exercise Capacity**<br>Bruce protocol<br>3. **Motor performance**<br>Movement	• Findings showed a decrease in exercise capacity (Mean SDS= −1.37 (1.09), *p* < 0.001) and motor skills of POMS patients (Mean SDS= 13 (35.1), *p* < 0.001), particularly in balance

	status, fatigue and health related quality of life in youth with MS and post-ADEM.				4.5 (2.3-5.9)		40.1 (11.4-63.5)	Assessment Battery for Children second edition (MABCII)<br>4. **Health-related quality of lif**e<br>Pediatric quality of life inventory 4.0 (PedsQl-HRQoL)	subscales (Mean SDS= 12 (32.4), *p* < 0.001).<br>• Further, decreased exercise capacity was correlated with decreased participation in organized sports (r = 0.365, *p*=0.034).
Zafar et al. 2012 [48]	To examine if POMS patients have more sleep disturbances, fatigue, and daytime sleepiness compared to controls.	Case-control	132	**Case**<br>30<br>**Matched Control**<br>52<br>**Historic Control**<br>52	**Case**<br>16.10 (1.37)<br>**Matched Control**<br>16.10 (1.71)<br>**Historic Control**<br>10.40 (14.45)	**Case**<br>73<br>**M Control**<br>65<br>**Historic Control**<br>77	2.6 (2.4)	1. **Fatigue**<br>PedsQL MFS<br>2. **Sleep quality**<br>Adolescent Sleep-Wake Scale (ASWS)<br>2. **Sleep hygiene**<br>Adolescent Sleep Hygiene Scale<br>3. **Daytime sleepiness**<br>Modified Epsworth Sleepiness Scale (mESS)	• Individuals with POMS were found to have better sleep hygiene, particularly in relation to sleep stability (*p*=0.0052), greater frequency of adherence to a usual sleep time throughout the week, and also less daytime sleepiness than controls (p=0.0061).
Carroll et al. 2016 [49]	To explore experiences of fatigue in paediatric MS and gain insight into how POMS youth and their parents deal with fatigue.	Qualitative methods were employed using in-depth semi-structured interviews.	28	**POMS**<br>15<br>**Parents of POMS youth**<br>13	*Median (range)*<br>**POMS**<br>15.2 (9-18)<br>**Parents of POMS youth**<br>46.8 (32-52)	**POMS**<br>53<br>**Parents of POMS youth**<br>85	*Median (range)*<br>2.9 (1-11)	Experience of fatigue and how it relates to sleep patterns.	• Children with POMS reported that daytime fatigue often led to napping, which disrupted their sleep patterns and led to poor sleep quality.
